# An empirical study exploring pre-service teachers’ profiles and their prospective ICT integration: is it a matter of attitudes, self-efficacy, self-concept or concerns?

**DOI:** 10.1007/s40692-022-00254-8

**Published:** 2022-12-24

**Authors:** Marcela Pozas, Verena Letzel, Julia Frohn

**Affiliations:** 1 Professional School of Education, Humboldt-Universität zu, Berlin, Germany; 2 School of Psychology, University of Monterrey, San Pedro Garza García, Mexico; 3 Section for Teacher Education and Research, Universität Trier, Trier, Germany; 4 Inclusive Research, Goethe-Universität Frankfurt, Frankfurt, Germany

**Keywords:** Pre-service teachers, Attitudes towards ICT, ICT self-efficacy, ICT self-concept, ICT concerns

## Abstract

Empirical evidence has revealed that affective-motivational variables such as pre-service teachers’ attitudes, self-efficacy, self-concept and concerns play a key role in their pedagogical decisions regarding whether and how to integrate technology within their classroom practices. However, there is still little research on interaction between pre-service teachers’ affective-motivational variables and their resulting impact on their ICT integration. By means of hierarchical cluster analysis, this study examines the relationship between pre-service teachers’ internal variables of ICT attitudes, self-efficacy, self-concept and concerns and its resulting effect on their future ICT in-class integration. A total of 155 pre-service teachers in Germany participated voluntarily in the study. The results revealed two distinct and opposite pre-service teachers’ profiles based on the four internal variables explored: ICT attitudes, self-efficacy, self-concept and concerns. In addition, the findings reveal no significant associations between the teacher profiles, age, teaching programme (school track), and more interestingly, amongst gender. Lastly, the results also indicate that the two pre-service teacher clusters significantly differ in their prospective ICT integration. An in-depth discussion, limitations as well as practical implications are presented in the paper.

## Introduction

It is without a doubt that technology-related factors (e.g. access to technological resources and devices) (Tondeur et al., 2017) as well as teachers’ digital competences Hatlevik & Hatlevik, 2018) are considered significant and relevant elements associated to in-service and pre-service teachers’ integration of information and communication technologies (ICT) in their teaching practice (Tiede et al., 2015). However, the meaningful integration of ICT in the classroom does not solely depend upon such factors. A variety of models have emphasized the importance of in-service and pre-service teachers’ affective-motivational factors on their use of ICT in classrooms (Jenßen et al., 2021). For instance, the ‘Will, Skill, Tool’ (WST) model incorporates affective-motivational variables such as attitudes and self-efficacy (Knezek & Christensen, 2016). Within the technology acceptance model, in-service and pre-service teachers’ attitudes towards ICT are also considered as essential factor for the decision and integration of ICT (Davis, 1989). Another example is Ertmer’s (1999) barriers to technology integration framework which identifies crucial factors that can either limit or foster teachers’ ICT implementation efforts: (a) external (first-order) variables, and (b) internal (second-order) variables. External variables are related to issues concerning access to technology, policy and training. However, even if these variables are present, “teachers do not automatically use technology to achieve advocated meaningful outcomes” (Ertmer, 1999, p. 51). As a result, it is necessary to take into consideration the impact of internal (second-order) variables on teachers’ ICT integration. Internal (second-order) barriers are teachers’ attitudes (Seufert et al., 2021), self-concept (Schweizer & Horn, 2014), self-efficacy (Hatlevik & Hatlevik, 2018) and concerns (Jogezai et al., 2018).

There is extensive evidence that has revealed that such internal variables play a key role in their pedagogical decisions regarding whether and how to integrate technology within their classroom practices (Petko, 2012; Seufert et al., 2021; Gil-Flores et al., 2017; Eickelmann, 2011; Hämäläinen et al., 2021; Tondeur et al., 2020; Pozas, 2021). Moreover, empirical studies have indicated that such variables are strong predictors of teachers’ ICT use integration (Krause et al., 2017; Seufert et al., 2021). Nevertheless, even though there is extensive research on such in-service and pre-service teachers’ attitudes, self-efficacy, self-concept and concerns regarding ICT, there is still little research on their interaction and their resulting impact on ICT integration (Hatlevik & Hatlevik, 2018).

Given the centrality and importance of internal variables, it appears meaningful and necessary to explore the interrelations between pre-service teachers’ internal variables and examine their link to their prospective ICT use in order to identify specific factors that can inform teacher training. Moreover, pre-service teacher training plays a crucial role in promoting the integration of ICT into classrooms (Botturi, 2019), thus it is of upmost importance to derive content for teacher training curricula from relevant scientific literature and research in order to ensure that pre-service teachers are specifically prepared for their prospective teaching profession (Tiede, 2020). Therefore, in this article, we explore the associations between pre- service teachers’ attitudes, self-concept, self-efficacy and concerns in using ICT for instructional purposes by means of cluster analyses. In the following sections, a description on the theoretical background as well as recent empirical findings are presented and followed by the research aims leading the study.

## Theoretical background

### Attitudes towards ICT

AJZEN (2005) defines attitudes as dispositions to respond favourably or unfavourably towards an object, person or event. Attitudes can be inferred from an affective, behavioural and cognitive component response towards the object of a specific attitude (Ajzen, 2005). They are considered to be a determinant of behavioural intention (Ajzen, 1991). As they are strongly related to actions (Haddock & Maio, 2014), they play a considerable role in teachers’ classroom practices (Baumert & Kunter, 2006; Schaarschmidt, 2005). In this context, many studies have focussed on measuring the impact of attitudes on ICT integration. Indeed, empirical research shows that favourable attitudes towards ICT influence teachers’ technology integration (Knezek & Christensen, 2016; Petko, 2012; Seufert et al., 2021). Likewise, research has found that attitudes towards ICT have a strong positive relation with the intention to use ICT in class (Celik & Yesilyurt, 2013; Kreijn et al., 2013; Petko, 2012; Sang et al., 2010; Scherer & Teo, 2019). Additionally, Bas et al. (2016) argue that in-service teachers who hold less positive attitudes towards ICT commonly invest less effort into adopting ICT in their instructional practices. A more recent study by Gretter & Yadav (2018) indicate that pre-service teachers hold mostly positive attitudes towards ICT and thus consider the use of ICT for educational purposes to be of great importance.

### ICT self-efficacy

Woolfolk (2004) defines self-efficacy as an individual’s belief in his or her ability to manage and handle situations. ICT Self-efficacy has been positively related to an individual showing effective coping behaviours when faced with computer-related difficulties. Recent findings show that teachers, who perceive higher levels of self-efficacy, experience less ICT-related anxiety and stress (Dong et al., 2020). Previous research has indicated that teachers’ self-efficacy towards ICT use plays an important role in how they integrate ICT into their instruction (Gil-Flores et al., 2017). Moreover, according to Sang et al. (2010), pre-service teachers’ ICT self-efficacy predicts their prospective computer use in education.

### ICT self-concept

Whilst teachers’ self-efficacy and self-concept are considered two important competence beliefs (Yeung et al., 2014), these two differ in multiple ways (Bong & Clark, 1999; Bong & Skaalvik, 2003). Whereas self-efficacy is measured by context-specific assessments, focuses on whether an individual has the capability to reach a future-oriented objective and is a malleable variable, self-concept, on the other hand, heavily relies on social comparison holding stronger evaluative implications, is past-oriented and relatively stable (Zhu et al., 2018).

Schavelson et al. (1976) define self-concept as an individual’s self-perception of his or her abilities in a specific domain. Thus, based on the theoretical framework and empirical research on self-concept, Schauffel et al. (2021) describe ICT self-concept “as an individual’s mental representations and evaluations of their own competences in dealing with ICT” (p. 100,149). In this sense, a teachers’ ICT self-concept can be broadly defined as teachers’ perceptions of their own teaching effectiveness when using ICT for teaching purposes. Teachers’ self-concept is a key variable that can have a positive impact on a teacher’s behaviour and supports the development of teachers’ professional identity (Helmke & Weinert, 1997; Roche & Marsh, 2000; Terhart, 2000). However, despite the importance of a teacher’s self-concept, research into this variable is still scarce (Lohbeck et al., 2018; Schweizer & Horn, 2014), in particular within the topic of ICT usage. To the best of our knowledge, only two studies could be identified that related to the topic. For instance, Schweizer & Horn (2014) explore pre-service and in-service teachers’ ICT self-efficacy, attitudes, beliefs and self-concept, and their relation to their ICT use for educational purposes. Interestingly, the findings indicate that both pre-service and in-service teachers hold higher levels of ICT self-concept, however, pre-service teachers’ ratings are slightly higher. Additionally, regression analysis indicates that only for the case of in-service teachers, their ICT self-concept predicted significantly their ICT use in class. Interestingly, the study by Lohbeck et al. (2018) shed similarly results: Pre-service teachers reported a significantly higher ICT self-concept than in-service teachers. According to the authors, these results may result from the fact that the current generation of pre-service teachers is commonly more familiar with ICT use. Finally, although self-concept is considered to be relatively stable (Zhu et al., 2018), a recent study by Rothland & Straub (2018) has indicated that pre-service teachers’ ICT self-concept significantly increased after a practical internship.

### ICT concerns

Whilst teachers’ attitudes, self-efficacy and self-concept are important predictors of success or failure of ICT integration, their concerns about ICT and media are equally important. Findings from the 2013 ICILS study (Fraillon et al., 2019), and more recently from the 2018 ICILS study (Rath & Delere, 2020), revealed that, in an international comparison, German teachers reported having high levels of concerns about ICT use. In detail, the study indicates that teachers’ concerns are related to potential organizational problems, possible distractions of students and the risk of copying content from the internet. Against this context, Fraillon et al. (2019) argue that this could be a reason for the below-average use of ICT in the classroom. Such a finding is also congruent with what others have found (e.g. Lorenz et al., 2019).

Important to highlight is that empirical research has also indicated that teachers’ concerns have a significant and negative impact on their attitudes (Yada & Savolainen, 2017). Thus, it can be argued that teachers who have relatively positive attitudes towards ICT integration are likely to have lower degrees of concerns about it or vice versa.

### Research aims

In light of the aforementioned theoretical background and outcomes of previous studies, it seems meaningful to explore the interrelations of pre-service teachers’ internal variables. Accordingly, this study seeks to investigate pre-service teachers’ profiles based on their ICT attitudes, self-concept, self-efficacy and concerns. Furthermore, it aims to analyse whether such pre-service teachers’ profiles differ with respect to their prospective (self-reported) ICT integration. Exploring pre-service teachers’ profiles could shed light on the differential learning developments of pre-service teachers during their teacher training (König, 2017), or identify content in teacher education that is necessary for developing their professional knowledge (Høgheim & Federici, 2020). This is of particular importance for the first phase of the German teacher education (Rösler et al., 2018).

With this background, this study’s research questions are:

1. What are pre-service teachers’ profiles of internal (second-order) variables?
2. Does pre-service teachers’ prospective ICT use for educational purposes differ amongst these profiles?

## Method

### Participants and procedure

To recruit participants, an invitation with a link to an online survey was sent to pre-service teachers, which were attending different teacher education courses and lectures. Participation took around 15 min and was completely voluntary and anonymously. The online survey started by asking participants for their consent. After consent was given, participants were asked for general demographic information, and was followed by the subscales which are included in this study.

Data were collected from 155 initial teacher education students at two public universities in Germany. Although the present study has a relatively small sample size, it still affords enough power for cluster analyses, as it assumes a detectable distance between centroids (Δ = 4), and a minimum group size of 25 participants per cluster or subgroup Dalmaijer et al. (2022). The mean age of the sample was 25.69 years (SD = 3.88 years). Please refer to Table 1 for the sample’s general demographic information. Data was collected during the 2020 summer and 2021 winter semester.

**Table 1:** Sample general demographic characteristics

Demographic characteristics		Percentage
Age	20–25 years	58.4%
	26–30 years	32.9%
	31–35 years	5.8%
	36–40 years	1.2%
	41–48 years	1.2%
Gender	Male	21%
	Female	79%
Study level	Bachelor studies	24%
	Masters studies	76%
Teacher training programme	Advanced secondary school	54%
	Intermediate secondary school	40%
	Special education	2%
	Missing	4%
Total		155

### Instruments

#### Demographic information

Firstly, demographic information was collected. This included information about gender (dummy coded: male = 1; female = 2), age, the number of semesters in the teacher education programme, the school track programme and school internships. The participants were also asked to indicate whether they had already attended seminars or courses on the use of digital media in school during their studies.

#### Pre-service teachers’ internal (second-order) variables

Pre-service teachers’ internal (second-order) variables were measured with an instrument developed by Tappe (2017) that stems from the work by Nistor et al. (2012). This instrument consists of four subscales: (1) attitudes towards ICT, (2) ICT self-concept, (3) ICT self-efficacy and (4) ICT concerns. Table 2 presents each of the subscales, item examples and reliabilities. As it can be observed, Cronbach’s alpha computed in IBM SPSS Statistics 27 was above 0.80 in all four dimensions, suggesting that the reliability seems adequate.

**Table 2:** Subscales used in the study

Subscale	Item example	Likert-scale	Cronbach’s *α*
Attitudes towards ICT (4 items)	I am happy when I can make use of digital teaching elements in my classes	1 = does not apply to 4 = applies completely	α = .87
ICT self-concept (6 items)	Compared to my colleagues, I feel competent when planning classes based on media didactics		α = .94
ICT self-efficacy (5 items)	I can plan a lesson including digital teaching elements even if I have only a limited amount of time to use them		α = .86
ICT concerns (4 items)	I have concerns about using digital media as teaching and learning tools		α = .80

#### Prospective computer use for educational purposes

Pre-service teachers’ prospective computer use for educational purposes was measured using an adapted version of the *Prospective Computer Use Scale* by Sang et al. (2010). Following a combined technique, the scale was translated into the German language (Cha et al., 2007). Thus, a bilingual translator blindly translated the questionnaire from English to German, and afterwards a second bilingual translator independently back-translated the instrument from German to English. Finally, the two versions of the questionnaire (German language and back-translated English version) were compared for equivalence. Two of the authors served as consultants during this process and supported by clarifying the meaning of items for translations. The adapted German version of this scale has been previously used in a study by Pozas (2021) and showed satisfactory instrument quality. The scale consists of 10 items based on a 3-point Likert scale ranging from 1 (*not at all interested*) to 3 (*very interested*) (e.g. ‘I would use the computer to assist with the differentiation or implementation of individual learning plans’; *α* = 0.85).

### Data analysis

Statistical analyses were conducted in IBM SPSS Statistics 27. Firstly, descriptive analyses and one sample *t* tests were performed to explore the data. Afterwards, a two-step cluster analysis was conducted in order to identify and group participants who have similar scores and to ensure the reliability of the clusters (Field, 2013). The first step consisted of a hierarchical cluster analysis using Ward’s method and squared Euclidean distance to identify the number of possible profiles of teachers (Hair et al., 1998; Yim & Ramdeen, 2015). The clustering variables were the four scales stemming from the work by Tappe (2017): attitudes towards ICT, ICT self-concept, ICT self-efficacy and concerns about ICT. The second step consisted of a k-means procedure to assign pre-service teachers to their profile and was followed up by an additional discriminant analysis in order to validate the number of clusters. Further analyses included chi-square tests of association in order to examine the relationship between the affiliation with the particular cluster and the school type in which pre-service teachers were currently enrolled. Lastly, Mann-Witney nonparametric test were conducted to explore potential mean differences between the clusters on the prospective computer use for educational purposes were analysed.

## Results

### Descriptive results

Before exploring the potential pre-service teacher profiles, descriptive analyses of the four subscales were undertaken by analysing means and standard deviations of each scale. Overall, as seen from Table 3, pre-service teachers’ attitudes towards ICT, and ICT self-concept were the variables with the highest scores, whereas ICT self-efficacy and ICT concerns had the lowest scores. A one sample t-test analysis revealed that pre-service teachers’ ratings of attitudes towards ICT [*t*(154) = 8.78, *p* < 0.001, Cohen’s *d* = 0.83] was significantly higher than the theoretical mean of the scale (3). In contrast, pre-service teachers’ ratings for both ICT self-efficacy [*t*(154) = − 4.63, *p* < 0.001, Cohen’s *d* = 0.83] and ICT concerns [*t*(154) = − 13.23, *p* < 0.001, Cohen’s *d* = 0.89] were significantly lower than the theoretical mean. Concerning pre-service teachers’ ICT self-concept, no significant difference to the theoretical mean was found. Such results imply that pre-service teachers hold positive attitudes and low concerns towards the use of ICT for educational purposes, however, lack confidence to appropriately plan and implement a lesson as well as manage a classroom when using ICT.

**Table 3:** Descriptive statistics of the variables understudy

Variable	*N*	Min	Max	*M*	*SD*
ICT prospective use	155	.00	3.00	2.42	.44
Attitudes towards ICT	155	.00	5.00	3.59	.83
ICT self-concept	155	.00	5.00	3.03	.93
ICT self-efficacy	155	.00	4.80	2.69	.93
ICT concerns	155	.00	4.25	2.06	.89

### Pre-service teacher internal (second-order) profiles: cluster analysis

**Fig. 1:**
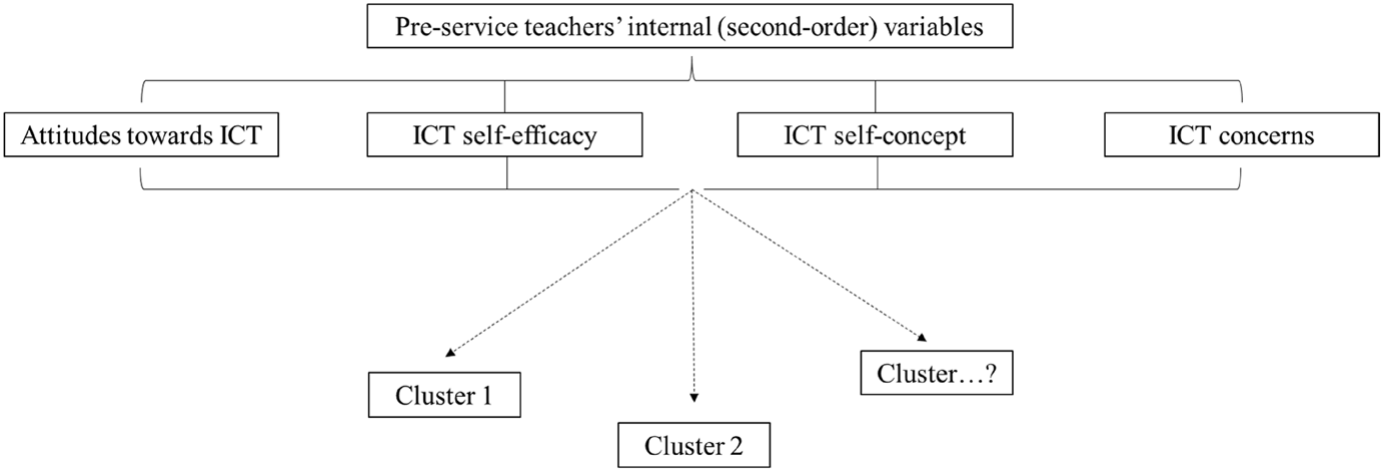
Study design model

As a first step, a hierarchical cluster analysis was performed to distinguish clusters between pre-service teachers based on the four internal (second-order) ICT barriers. As seen from Fig. 1, the dendrogram from the hierarchical cluster analysis indicated that there were several options for 2- or 3- clusters. Based on these two possible solutions, a k-means cluster analysis was conducted based on the 3-cluster solution. However, results revealed that one cluster had only two cases. Therefore, a second k-means cluster analysis was conducted based on a 2-cluster solution to assign the pre-service teachers into their profile.

As a final step, a discriminant analysis was performed where one discriminant function was identified. This function showed a canonical correlation of *R* = 77.3% (eigenvalue = 1.49; Wilks Lamdba = 0.40; *p* < 0.001; explained variation 100%). A total of 97.4% of the cases were correctly classified, and thus 2.6% were reassigned accordingly. The final clusters are composed as follows: cluster 1 included 119 pre-service teachers (77%), whereas cluster 2 included 36 teachers (23%). As shown in Table 3, one-way ANOVA with post-hoc analyses indicated that all four scales (attitudes towards ICT, ICT self-concept, ICT self-efficacy and ICT concerns) significantly varied within clusters, and therefore, these profiles were valid (see Table 4).

**Table 4:** Descriptive statistics and one-way ANOVA of the pre-service affective and motivational domains between profiles

Domain	Cluster 1		Cluster 2		*F(1,53)*	*η*^*2*^
	*M*	*SD*	*M*	*SD*		
Attitudes towards ICT	3.85	.60	2.74	.94	71.30**	.32
ICT self-concept	3.39	.66	1.83	.68	151.02**	.50
ICT self-efficacy	2.98	.60	1.76	.77	98.83**	.39
ICT concerns	1.92	.78	2.51	1.06	13.34**	.08

**p < .01

### Description of the clusters

**Fig. 2:**
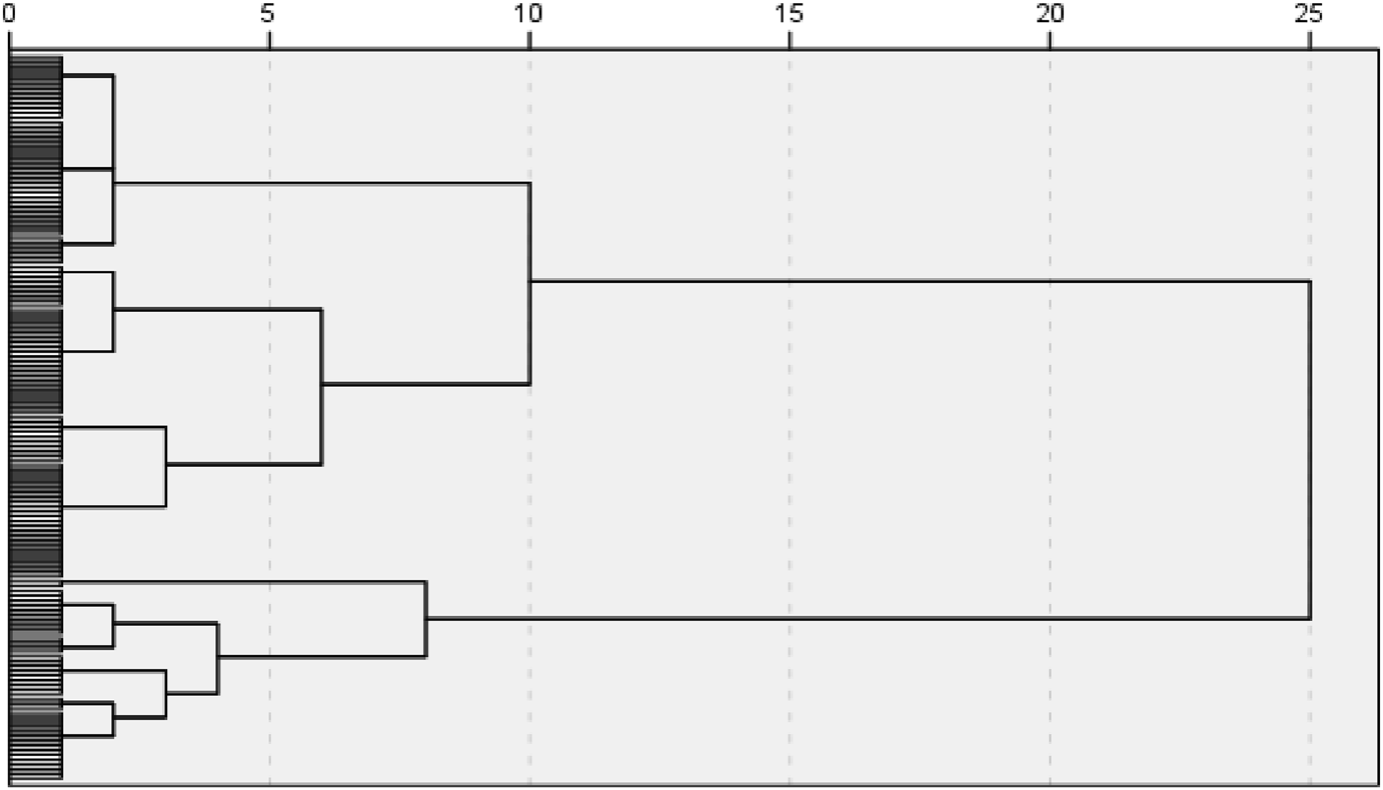
Dendrogram

The following section offers a description of the three clusters and Table 5 presents a summary of the clusters, whilst Fig. 2 visually presents the pre-service teacher profiles:

1. Cluster 1 *‘Can-do-ICT type’*: Pre-service teachers within this cluster scored significantly higher in the subscale of attitudes towards ICT, whereas significantly lower in ICT concerns. With regards to their ICT self-concept and self-efficacy, pre-service teachers scores were about the average scale value. Hence, it can be assumed that pre-service teachers in this cluster have a positive view on the use of ICT within the classroom environment, consider themselves moderately capable and confident to be able to plan and incorporate ICT instruments or tools in their teaching practice, and have less worries about the didactical use as well as potential difficulties whilst teaching with ICT instruments or tools.
2. Cluster 2 *‘Discouraged-ICT type’*: Pre-service teachers sorted into this cluster scored the lowest in both their ICT self-concept and self-efficacy, but about the average scale value concerning their attitudes towards ICT. Compared to cluster 1, these pre-service teachers reported significantly higher level of ICT concerns. Taken together, the mean values indicate that, although these pre-service teachers hold a neutral view on the use of ICT for their teaching practice, they do have slight worries and concerns about the didactical implementation of ICT in their classrooms. Additionally, and more importantly, they do not consider themselves capable nor confident to appropriately and meaningfully incorporate ICT in their teaching practice (see Table 5).

**Table 5:** Summary of the clusters

Cluster 1 ‘Can-do-ICT type’	Cluster 2 ‘Discouraged-ICT type’
High score: Attitudes towards ICT	High score: ICT concerns
Low score: ICT concerns	Low score: ICT self-concept and ICT self-efficacy
Average score: ICT self-concept and ICT self-efficacy	Average score: Attitudes towards ICT

**Fig. 3:**
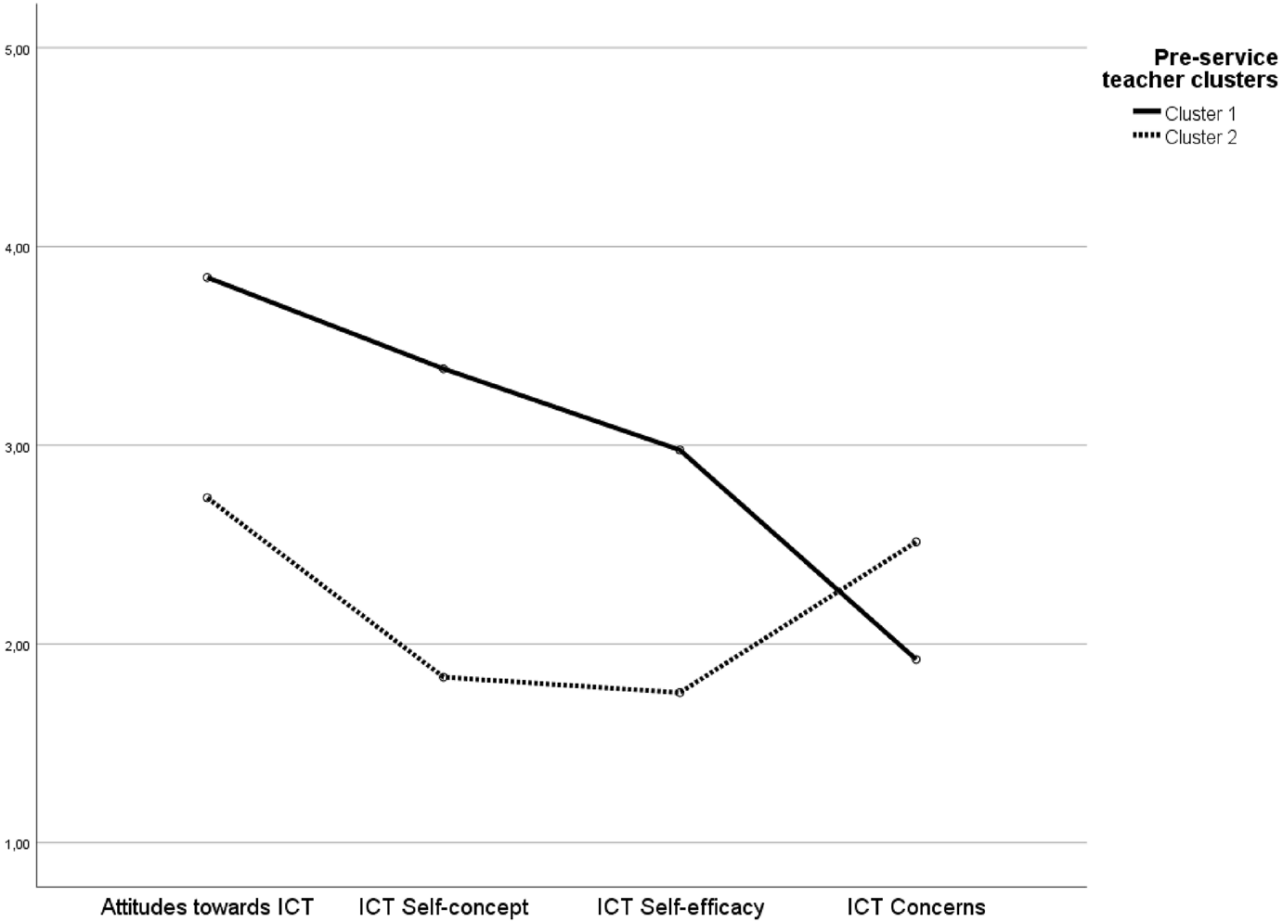
Pre-service teacher ICT profiles

Lastly, a multivariate analysis of variance (MANOVA) was conducted to explore whether the scores of the separate variables in each of the profiles differed between the clusters. MANOVA has been selected instead of running multiple ANOVAs as a means to prevent the risk of committing a Type 1 error and maintain the relationship between the variables (Field, 2013). The Wilks’ Lambda was revealed to be significant highlighting the differences between the clusters, [*F*(4,150) = 1026.47, *p* < 0.001, *partial η2* = 0.97]. Finally, chi-square tests of association were used to examine whether there was a relationship between the profiles and the demographic variables. The analyses showed no significant association between teacher profile, age, gender and teaching programme (school track) (See Fig. 3).

### Comparison of the pre-service teacher internal (second-order) profiles and their prospective ICT use

Given that more than 70% of the participants were sorted in cluster 1, it was decided to compare the pre-service teacher ICT profiles using the Mann–Witney nonparametric test (Field, 2013). Results from the analysis revealed that pre-service teachers’ prospective ICT use for educational purposes varied between the two clusters. In detail, it appears that pre-service teachers in cluster 1 (*Mdn* = 2.50) are more likely to make use of technology in their future instructional practice in comparison to pre-service teachers within cluster 2 (*Mdn* = 2.30), *U* = 1654, *z* = − 2.08, *p* < 0.05, *r* = − 0.17.

## Discussion

In-service and pre-service teachers’ internal variables such as ICT attitudes, self-efficacy, self-concept and concerns are crucial to a teachers’ teaching behaviour, such as the use ICT for teaching purposes (Ertmer, 1999; Krause et al., 2017; Seufert et al., 2021). However, despite the extensive research on teachers’ internal variables, there is still limited empirical research on their interaction and their resulting impact on ICT integration (Hatlevik & Hatlevik, 2018). Hence, this study aimed to gain deeper insight into the links between pre-service teachers’ internal variables by means of clustering analysis. Furthermore, the present study explored differences in pre-service teachers’ prospective ICT use for educational purposes across the clusters. The mean and standard deviation scores of pre-service teachers’ ratings suggest that they hold fairly positive attitudes towards ICT. Such result is consistent with previous studies on pre-service teachers’ attitudes towards ICT (Gretter & Yadav, 2018). This is an important finding as in-service and pre-service teachers’ attitudes play an important role in their teaching behaviours such as the integration of ICT in classroom practices (Krause et al., 2017; Seufert et al., 2021). Moreover, pre-service teachers’ concerns of their ICT use were relatively low. Previous studies have revealed mixed results on in-service teachers’ levels of concerns about ICT (Pepe, 2016; Puteh et al., 2011). However, Jogezai et al. (2018) discussed that teachers’ concerns depend on technical infrastructures (tablets, learning management systems, etc.) as well as the fact that the emergence of concerns is developmental and thus, can be divided into categories that centre on self, task and impact. In this sense, in-service teachers might rate their level of concern differently when relating to specific ICT devices and stages of usages. Within this study, the instrument used to assess pre-service teachers’ concerns cannot provide insights into whether pre-service teachers’ concerns differentiate across different categories, and thus, these results should be considered with caution. For instance, in their study, Jogezai et al. (2018) found that teachers are mainly preoccupied with the task itself (when using ICT). Thus, further research is strongly suggested to use instruments that consider the multidimensionality of the construct as well as interviews that allow an in-depth exploration that could provide more fine-grained information. Likewise, and in line with past studies, self-efficacy towards ICT use was rated as rather negative by the sample. However, this result is consistent with previous research that has indicated that pre-service teachers perceive themselves as less self-efficacious with regard to ICT use for instructional purposes (Valtonen et al., 2021). This is an interesting result given that past research has indicated that pre-service teachers feel rather positive with regards to their efficacy teaching with ICT (Pozas, 2021). Nonetheless, researchers have argued that pre-service teachers might consider themselves ‘efficacious’ based upon the general or recreational use of ICT (Maderick et al., 2015) rather than on the professional requirements to “create good digitally-supported learning environments that foster high-quality learning” (Jenßen et al., 2021, p. 185). With this context, it can be assumed that pre-service teachers feel efficacious based on their familiarity with ICT resources and tools, but still do not feel sufficiently prepared in order to incorporate it as a crucial factor in their everyday teaching practice in such a way that promotes meaningful learning activities (Gretter & Yadav, 2018; Jenßen et al., 2021). Lastly, pre-service teachers’ ICT self-concept was not significantly over the theoretical mean, but rather neutral. A study by Schweizer & Horn (2014), in contrast, suggested that pre-service teachers’ ratings were relatively high. However, the authors used an adapted version of the academic self-concept scale (SESSKO) (Dickhäuser et al., 2002). Given that this adaptation was more related to the teaching profession rather than specifically for ICT, this could inherently account for the discrepancies amongst studies (see Fig. 3).

Second, the study’s results help gain a deeper insight into the mechanisms and interrelationships between internal variables related to ICT by clearly categorizing two different teacher profiles. Cluster 1 and 2 can be seen as opposites (‘*Can-do-ICT type*’ vs. ‘*Possibly-can’t-do-ICT type*’), as Cluster 1 scored significantly higher in ICT attitudes, self-concept and self-efficacy, whereas Cluster 2 scored the lowest, and vice versa with internal variable of ICT concerns. Pre-service teacher within Cluster 1 appear to not only think positively about using ICT for teaching purposes, they also perceive themselves efficacious to pro-actively use ICT in their instruction. However, in Cluster 2, pre-service teachers feel less efficacious and positive, which inherently is reflected in their concerns regarding the potential difficulties and challenges that ICT might bring to a lesson.

In addition, the results indicated no significant associations between the teacher profiles, age, teaching programme (school track), and even more interesting, amongst gender. Numerous studies have indicated gender differences when it comes to internal variables related to ICT usage, for instance, that females hold less positive attitudes towards ICT than males (Tondeur et al.,^ 2016^). In contrast other studies have not indicated such differences (Gebhardt et al., 2019; Hatlevik & Hatlevik, 2018). However, it is important to highlight that previous international comparisons studies have revealed gender differences only when conducting country-level analyses (Drossel et al., 2016; Gebhardt et al., 2019).

When exploring differences across the pre-service teacher profiles, results revealed that teachers allocated within the cluster 1 are more likely to make use of technology in their future instructional practice than pre-service teachers within cluster 2. This result goes in line with previous studies that have indicated that in-service and pre-service teachers holding more positive ICT attitudes (Krause et al., 2017; Seufert et al., 2021), higher levels of self-efficacy and self-concept (Baturay et al., 2017; Gil-Flores et al., 2017; Hatlevik & Hatlevik, 2018; Schweizer & Horn, 2014; Tondeur et al., 2020) and lower concerns (Hao & Lee, 2017; Jogezai et al., 2018; Puteh et al., 2011) tend to integrate ICT into their classroom practice. Important to highlight is that most teachers were ascribed to Cluster 1 (77%), thus indicating that in the current sample pre-service teachers considered themselves positive, confident, capable and are willing to teaching with ICT. However, this does not mean ‘good news’. Teachers within Cluster 1 have average ratings scores of ICT self-efficacy and self-concept, and teachers within Cluster 2 have even lower scores. Hence, it can be assumed that teachers within both clusters, and in particular within Cluster 2, would incorporate ICT more frequently, if they had such internal variables far much positive developed. All in all, a low score on attitudes towards ICT, ICT self-efficacy and self-concept separately is a reason to integrate ICT to a lesser extent. A plausible explanation for this can be found within the theory of planned behaviour (Ajzen, 2020). Pre- and in-service teachers that hold a positive attitude towards the integration of ICT, as well as feel efficacious and confident, tend to have a higher intention of actually using ICT for instructional purposes.

Thus, the findings within this study emphasize the importance, relevance and potential that teacher education has on supporting and fostering pre-service teachers’ attitudes, self-efficacy, self-concept and concerns in order to prepare them for their prospective teaching profession.

### Practical implications

Taking the findings from this study together, it is possible to contribute several practical implications, in particular for teacher training programmes. First, teacher education should develop and foster pre-service teachers’ attitudes, self-efficacy and self-concept concerning ICT practices (Hans et al., 2017). Additionally, it should provide the appropriate training of pre-service teachers to use ICT as a teaching tool (Agyei & Voogt, 2010; Sang et al., 2010; Tondeur et al., 2012; Urez et al., 2018). In order to achieve this, it is necessary that at a policy level, teacher training institutions ensure the inclusion of obligatory courses that address—theoretically but also practically—the use of ICT for teaching purposes within real classrooms. Although within the theoretical background it is described that many institutional bodies such as the KMK in Germany, have included the development of ICT competences within their teacher education standards, surprisingly, various recent studies have revealed that ICT plays a minor role in teacher training programmes (Tiede, 2020; Tiede et al., 2015). Additionally, pre-service teachers should be able to observe ICT integration during their teaching internships, practice using ICT and collaborate with their peers in authentic scenarios within action-oriented courses (Rubach & Lazarides, 2020; Tondeur et al., 2012). To this end, schools and universities should team up to enable pre-service teachers to work together and be mentored by in-service teachers in real ‘teaching’ situations (Hobbs et al., 2011; Tondeur et al., 2018). Lastly, teacher educators play a crucial role, as they serve as role models and must possess the pedagogical knowledge and skills that they want their students to acquire (Urez et al., 2018). In other words, teacher educators should not only deliver content, but should also teach and model technology use. As shown by previous research, every effort can be effective in fostering the attitudes, self-efficacy and self-concept of pre-service teachers and developing their competences (Botturi, 2019; Rothland & Straub, 2018; Valtonen et al., 2021).

## Limitations and further research

The present study followed convenience sampling method. Although this is a common research strategy, it possesses nevertheless several disadvantages such as the fact that the results obtained from such samples have generalizability only to the sample understudy (Bornstein et al., 2013). Additionally, the study was carried out at two universities in Germany. According to Tiede (2020), the integration of ICT-related courses into teacher training programmes in Germany significantly differs from state to state. For instance, in some universities in certain states, teacher training programmes include obligatory ICT courses, whereas in other universities such courses are elective courses which students can decide to take voluntarily. In the present study, differences across the universities were not conducted, and thus cannot explore potential differences between clusters arising from the different teacher education programmes. Consequently, generalization of the results to other universities or teacher training institutions is not possible. Similarly, the data analysed in this study was collected in only one country, thus, it cannot be assumed that the results are representative for other countries. Considering that teacher education in each country has its distinctive features, which are shaped by specific ICT competency models (Tiede, 2020), it is of upmost importance to conduct international comparative research. In addition, considering that normative documents, such as ICT educational policy papers, teacher education standards, competency model papers, “articulate in-class digital ability development” (Bolaños & Pilerot, 2021, p. 1), it would be important to explore how such normative documents influence how certain learning and training experiences are perceived and understood by both pre- and in-service teachers.

Another limitation is that the study uses pre-service teachers’ self-reports. Hence, such responses can inherently be sensitive to overestimation, underestimation, or socially desired answers. As suggested by Maderick et al. (2015), further research should follow a more holistic approach and include other methods such as observations and/or interviews. Moreover, given that different methods of clustering analysis could provide different results (Field, 2013), it is necessary for further research to test such structure in other German pre-service teachers and conduct qualitative interviews with participants as a means to confirm and validate the link between the respondents and the cluster they were ascribed to (Vanslambrouck et al., 2018).

The study explored the impact of internal variables on teachers’ ICT integration and did not focus on competences. Teachers’ digital competences are also an important determinant of ICT use in education (Tiede et al., 2015). Therefore, further research should also strongly incorporate such variable when explore pre-service teachers’ ICT profiles. Further limitations of the study relate to the small cluster size (30% of the participants were sorted into cluster 2) which could be inherently due to the small sample size and the higher percentage of female participants. However, the sample distribution is representative of the female and male student population in teacher education (Stephan et al., 2019).

## Conclusion

Teachers play a crucial role in the pedagogically sound and effective incorporation of ICT into classrooms. Besides technology-related factors and ICT competences, in-service and pre-service teachers’ affective-motivational variables such as attitudes towards ICT, ICT self-efficacy, ICT self-concept and concerns towards ICT are essential factors that can hinder or foster whether and how ICT is integrated into a classroom. Although various research has explored such variables, their interrelations remain rather unexplored. Thus, through a cluster analysis, the results show that pre-service teachers’ affective-motivational variables have a complex and dynamic interplay, which inherently determines the extent to which a pre-service teacher will integrate ICT into their future in-class teaching. These findings are interesting for both, teacher professionalization and teacher education as they emphasize the urgency to invest efforts into supporting pre-service teachers’ affective-motivational variables and not only their ICT competency, as a lack of agency and high concerns can be together pitfalls for why pre- and in-service teachers do not succeed in successfully integrating ICT in their teaching.

Lastly, given the current Coronavirus crisis, it can be concluded that, now more than ever, pre-service teachers need to be provided with well-rounded ICT training based on authentic experiences, preparing them for their future role as teachers who can effectively include ICT in their daily teaching practice.
